# Pain and Cognitive Concerns Among Breast Cancer Survivors: The Mediating Role of Substance Use Coping

**DOI:** 10.1002/cam4.71204

**Published:** 2025-09-29

**Authors:** Yesol Yang, Alai Tan, Leorey N. Saligan, Diane Von Ah

**Affiliations:** ^1^ Center for Healthy Aging, Self‐Management and Complex Care The Ohio State University College of Nursing Columbus Ohio USA; ^2^ Cancer Control Program The Ohio State University‐ James: Cancer Treatment and Research Center Columbus Ohio USA; ^3^ Rutgers University, School of Nursing Newark New Jersey USA

**Keywords:** breast cancer survivors, cognitive function, coping, pain, substance use

## Abstract

**Context:**

Bodily pain and cognitive concerns are both prevalent and share similar underlying mechanisms. A few studies have suggested that pain and cognitive concerns may be linked through substance use coping; however, this relationship remains unclear, particularly among breast cancer survivors (BCS). Gaining a clearer understanding of this relationship is critical, as it could inform survivorship care plans for BCS.

**Objectives:**

This study aims to investigate whether bodily pain is related to cognitive concerns among BCS and to further explore whether this relationship is mediated by substance use coping.

**Methods:**

This study is a sub‐analysis of data obtained from a larger cross‐sectional study. Bodily pain was assessed using the Short‐Form Health Survey, and cognitive concerns were measured using the Patient‐Reported Outcomes Measurement Information System. Substance use coping was assessed using two items from the Brief Coping Orientation to Problems Experienced Inventory. Path analysis was used to examine the relationships among bodily pain, substance use coping, and cognitive concerns.

**Results:**

Higher levels of bodily pain and higher use of substance use coping were associated with greater cognitive concerns. Although the relationship was weakly related, we found that bodily pain was associated with substance use coping, and that substance use coping mediated the association between bodily pain and cognitive concerns.

**Conclusion:**

These findings highlight the importance of regular pain assessments to help prevent future behavioral and cognitive consequences. Moreover, efforts to screen for and manage both pain and substance use may ultimately help prevent cognitive problems during survivorship.

## 1 Introduction

Pain and cognitive concerns are common complaints of breast cancer survivors (BCS) [1]. Pain is reported by 30%–40% of BCS after active cancer treatments [2, 3], and up to 75% of BCS report cognitive concerns [4]. BCS report varying types and locations of pain resulting from cancer treatment [5]. Some BCS who underwent surgery report chronic pain primarily in the affected breast and adjacent upper limbs (axilla, arms, shoulders) [6, 7, 8]. Others treated with chemotherapy and/or radiation report persistent musculoskeletal pain as well as more diverse painful sites, including upper limbs, spine, and head [9, 10]. Such posttreatment bodily pain can result in negative mental, behavioral, and cognitive outcomes for BCS [2, 11, 12]. Cognitive concerns commonly reported by BCS are associated with negative outcomes in social relationships, work performance, medication adherence, and quality of life [13, 14, 15].

Although pain and cognitive problems are both prevalent and share similar underlying mechanisms, only a few studies have examined them as associated symptoms [11, 16, 17]. One large‐scale longitudinal study suggested pain is a predictor of cognitive dysfunction by showing that BC patients who had high levels of psychoneurological symptoms, including pain, at diagnosis demonstrated persistently lower cognitive function during survivorship [18]. A recent study revealed that pain and cognitive problems co‐occur during the cancer survivorship period [18] and further reported that both symptoms are related to coping strategies [18]. This study also indicated that patients with both moderate and severe levels of pain and cognitive problems tend to use disengagement coping, including substance use, although it did not clarify how this coping is related to pain and cognitive function [18].

Coping is defined as cognitive, emotional, and behavioral efforts that people make to manage stressful experiences [19]. Coping strategies can be broadly divided into adaptive and maladaptive strategies [20]. Adaptive strategies use healthy and effective ways to deal with stress (e.g., problem‐ and emotion‐ focused), while maladaptive strategies offer temporary relief but are ultimately harmful (e.g., substance use or denial) [20]. Given that pain and cognitive problems are distressing symptoms, the ways in which individuals cope with stress may be linked to pain and cognitive function, although this relationship remains unclear in cancer survivors. A recent study identified alexithymia, the impaired ability to recognize and express emotions, as a modifiable risk factor for the development of persistent pain following breast cancer surgery, independent of anxiety and depression [21]. Individuals with alexithymia are more likely to interpret symptom experiences as highly distressing, which may increase their reliance on drug use as a coping strategy [22].

Pain is an aversive experience associated with significant physical, psychosocial, and economic burden [23]. When experiencing pain (either sensory or affective pain), some individuals tend to use substances (e.g., alcohol or drugs) to numb difficult emotions and escape these distressing experiences [19, 24]. Studies have shown that pain drives individuals with chronic pain to consume substances [25, 26], and up to 74% report misusing substances [27, 28, 29]. Furthermore, several other studies have shown that substance use can negatively affect cognition as such use can change brain function and structure, resulting in cognitive deficits [30, 31]. Similar to these findings, some cancer studies found that alcohol or drug use negatively affects cognitive function in cancer survivors [32], and should be monitored during cancer survivorship [33, 34].

Together, findings suggest that pain seems to reinforce substance use as a coping strategy and that substance use coping is linked to cognitive function. However, these links are unclear, particularly among BCS. To address this gap in the literature, the present study investigated the mediating role of substance use coping in the association between bodily pain and subjective cognitive concerns among BCS. It was hypothesized that higher levels of bodily pain would be positively associated with greater subjective cognitive concerns and that this association would be mediated by substance use coping. Gaining a clearer understanding of this is critical because it will provide insights that can inform survivorship care plans for BCS.

## 2 Methods

### 2.1 Study Design and Participants

This study was a secondary data analysis of the cross‐sectional data obtained from individuals with breast and colorectal cancers to investigate cancer‐related cognitive impairment (ClinicalTrials.gov Identifier: NCT04611620). The Institutional Review Board at Indiana University and the Melvin and Bren Simon Comprehensive Cancer Center both gave their approval for the parent study. In the parent study, recruitment was conducted via breast cancer foundations which approved distribution and posted information about the study to their members (e.g., Facebook and online cancer resources website like Pink Ribbon Connection, Dr. Susan Love Foundation) [35, 36, 37].

For this current sub‐analysis, we used BCS' data from the parent study. Eligibility criteria for BCS were: (1) aged 21 years or older; (2) ≥ 6 months after completing adjuvant/neoadjuvant therapies for breast cancer (current Aromatase Inhibitors use at the time of enrollment was allowed); (3) having cancer‐related cognitive impairment; and (4) able to provide written consent and HIPAA authorization.

### 2.2 Data Collection

Eligible participants were given unique identifiers after obtaining informed consents virtually and were provided a secure HIPAA‐protected link to complete a series of survey questions. The participants' data on current age, race/ethnicity, marital status, prior educational history, current work status, income, diagnosis and clinical stage, and cancer treatments (chemotherapy, surgery, and/or radiation therapy) were all collected via the demographic and clinical forms (created by researchers). Details of the data collection procedures are available in previous publications [35, 36, 37].

### 2.3 Study Measures

Bodily pain was measured by two items of bodily pain from the Short‐Form Health Survey (SF‐36). The SF‐36 has been established as a comprehensive measure of general health that has shown reliability and validity in various populations including cancer patients [38]. In this analysis, we calculated the bodily pain score as the average of the two item scores on a 0–100 range, with higher scores representing higher pain. For this analysis, reliability for the SF‐36 bodily pain scale ranged from 0.80 to 0.94 [37].

Substance use coping was assessed by two items from the Brief COPE (Coping Orientation to Problems Experienced) Inventory [24]. These two items asked if participants have used alcohol or other drugs to feel better and to navigate life. Participants rated the extent to which they usually used each coping strategy when dealing with cancer‐related stress using a 4‐point Likert scale ranging from 1 (“I haven't been doing this”) to 4 (“I've been doing this a lot”). The substance use coping scale scores (range 2–8) were calculated by summing the two item scores, with high scores indicating greater use of substance use coping strategies. For this analysis, the reliability of the substance coping subscale was 0.82. Due to the highly screwed distribution of subscale score for substance use coping (median = 2.0, IQR = 2.0–3.0, range = 2–8), we used *z*‐transformed scores in the analyses.

Subjective cognitive concerns were assessed by the PROMIS (Patient‐Reported Outcomes Measurements Information) version 1.0 short‐form of the Cognitive Concerns scale, which contains 8 items [39]. The Cognitive Concerns items are worded to express concerns in the same cognitive areas. Two examples are “My thinking has been slow” and “I have had trouble shifting back and forth between different activities that require thinking.” Items use a 5‐point rating from 1 (“not at all”) to 5 (“very much”). The subjective cognitive concern scale scores (range 8–40) were calculated by summing the 8 item scores, with higher scores indicating higher cognitive concerns. The obtained scores can also be converted to standardized scores with a mean of 50 and a standard deviation of 10, based on a reference population. A higher T‐score generally indicates a greater level of cognitive concerns. For this analysis, the reliability of this subscale ranged from 0.80 to 0.83 [37].

### 2.4 Data Analysis

Descriptive statistics were used to summarize sample characteristics and their level of bodily pain, substance use coping, and cognitive concerns. Substance use coping was *z*‐score transformed to meet a normal distribution. Pearson correlation coefficients were used to examine the pairwise associations among bodily pain, substance use coping, and cognitive concerns. We conducted path analysis to explore the mediational role of substance use coping (mediator) in the association between bodily pain (predictor) and cognitive concerns (outcome). A percentile bootstrapping approach with 5000 samples was used to estimate the indirect (or mediation) effects of bodily pain on cognitive concerns via substance use coping. This model included one direct effect (the effect of bodily pain on cognitive concerns) and one indirect effect (the effect of bodily pain on cognitive concerns via substance use coping), both adjusted for age, comorbidities, and use of aromatase inhibitors. The analyses were conducted using SPSS 28.0 (SPSS Inc., Chicago, IL, USA). The mediational analysis was conducted using the SPSS PROCESS macro.

## 3 Results

### 3.1 Sample Characteristics

The 559 breast cancer survivor participants had a mean age of 55.8 years (SD = 12.0). The majority were White women (*n* = 525, 92%), non‐Hispanic (*n* = 529, 96.4%), currently married (*n* = 325, 64.6%), employed either full‐time or part‐time (*n* = 325, 58.2%), and having at least a secondary education (*n* = 511, 91.4%). Approximately 45% of survivors were diagnosed with stage II breast cancer, followed by stage I (*n* = 168, 30.1%) and stage III (*n* = 139, 24.9%). The most common treatments that participants received were chemotherapy (*n* = 511, 91.4%), surgery (*n* = 556, 99.5%), radiation therapy (*n* = 401, 71.7%), and an aromatase inhibitor (current and past use; *n* = 287, 51.3%). Approximately 30% of participants reported having one of the following medical problems: high blood pressure (*n* = 164, 29.3%), peripheral vascular disease (*n* = 150, 26.8%), or congestive heart failure (*n* = 149, 26.7%). Detailed demographic and clinical information of participating BCS are summarized in Table 1.

**TABLE 1:** Sample characteristics (*N* = 559).

Characteristics	*N* (%) or mean (SD)
Age, years	55.8 (12.0)
Ethnicity	
Hispanic	20 (3.6%)
Not Hispanic	529 (96.4%)
Race	
White	525 (92%)
Other	47 (8%)
Currently married	
Yes	361 (64.6%)
No	198 (35.4%)
Education	
< High school graduate	1 (0.2%)
High school graduate	47 (8.4%)
> High school graduate	511 (91.4%)
Employment status (full‐time or part‐time)	
Yes	325 (58.2%)
No	209 (37.3%)
Other/prefer not to answer	25 (4.5%)
Breast cancer stage	
I	168 (30.1%)
II	252 (45.1%)
III	139 (24.9%)
Chemotherapy	
Yes	511 (91.4%)
No	48 (8.6%)
Radiation therapy	
Yes	401 (71.7%)
No	158 (28.3%)
Surgery	
Yes	556 (99.5%)
No	3 (0.5%)
Aromatase inhibitor	
Never used	206 (36.9%)
Currently using	185 (33.1%)
Used in the past	102 (18.2%)
Don't know	66 (11.8%)

### 3.2 Correlation Analysis Between Pain, Substance Use Coping, and Cognitive Concerns

The sample had an average subjective cognitive concern score of 27.6 (SD = 7.6, range = 8–40). The average pain score was 46.3 (SD = 26.16, range 0–100), and substance use coping score was 2.67 (SD = 1.39, range = 2–8). Pair‐wise correlations are presented in Table 2. A higher pain score was associated with a greater substance use coping strategy (*r* = 0.13, *p* = 0.00), and substance use coping was positively associated with cognitive concerns (*r* = 0.14, *p* < 0.001). Additionally, higher pain was associated with higher cognitive concerns (*r* = 0.36, *p* < 0.001).

**TABLE 2:** Correlation among pain, substance use coping, and cognitive concerns.

	*N*	Mean (SD)	Correlation coefficient, *r*	
			Substance use coping, *z*‐score	Cognitive concerns
Bodily pain	530	46.3 (26.16)	0.13 (*p* = 0.00)^+^	0.36 (*p* < 0.001)^++^
Substance use coping, *z*‐score	530	0 (1.0)		0.14 (*p* = 0.001)^+^
Cognitive concerns	530	27.7 (7.6)	—	—

*Note:* Effect size: + small (*r* of 0.1–0.3), ++ medium (*r* of 0.3–0.5).

### 3.3 Indirect Effect of Pain on Cognitive Concerns via Substance Use Coping

**FIGURE 1:**
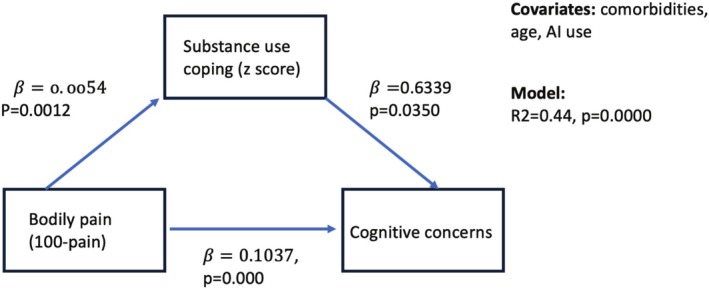
Path model results for body pain, substance use coping, and cognitive concerns. Standardized path coefficients (SE) were presented.

As noted in the path models with standardized estimates (Figure 1), higher pain was weakly but statistically significantly associated with greater substance use coping (*β* = 0.0054, *p* = 0.0012), and subsequently greater substance use coping was associated with greater cognitive concerns (*β* = 0.6339, *p* = 0.0350). From the path model, we further partitioned the total association (*β* = 0.11, SE = 0.01) between bodily pain and cognitive concerns into direct and indirect associations (see Table 3).

**TABLE 3:** Effects of pain on cognitive concerns.

	Effect	*β* (SE)	*p*
Standardized estimate	Total	0.11 (0.0115)	0.000
	Direct	0.10 (0.0115)	0.000
	Indirect (via substance use coping)	0.0034 (0.0019)	0.0003

## 4 Discussion

We found that higher bodily pain and higher substance use coping are associated with higher cognitive concerns, although there was a weak association between bodily pain and substance use coping. This could be because breast cancer patients involved in this study reported relatively lower bodily pain compared to other cancer survivor cohorts [40, 41], which could contribute to the failure in establishing a relationship with substance use coping. Although they are rather weakly related, it is an important initial finding that suggests a target for the prevention and treatment of substance use problems. According to the CANUE (Catastrophizing, Anxiety, Negative Urgency, and Expectancy) model, pain and substance use are hypothesized to interact in a positive feedback loop, resulting in the worsening of both conditions over time [25]. This model has further proposed pain as a critical motivator of substance use [25]. Along these lines, a recent review paper on cancer patients has also supported this model, suggesting that pain is associated with substance misuse [34]. Further studies are needed to include social determinants of health, a comprehensive assessment of pain, type and frequency of substance use, and current pain management (including opioid use) to better examine the pattern of substance use among BCS with pain, as its use has adverse outcomes.

Our results on the positive association between pain and cognitive concerns align with several cancer studies showing that pain severity and pain interference on daily functioning relate to cancer survivors' poor cognitive function [11, 42, 43]. The link between pain and cognition has been noted in some cancer studies; however, there remains a paucity of studies examining how they are related. Several studies suggested that at the neural level, there is a functional overlap in the pathways in the brain regions responsible for executive function and pain perception [44]. In addition, brain structural changes in the dorsolateral prefrontal cortex and anterior cingulate cortex were noted among those with chronic pain [44]. One review paper discussed the link between pain and cognition and explained that pain leads to the overactivation of microglia involved in regulating homeostasis in the brain, and such overactivation changes and remodels the brain network, resulting in cognitive impairment [45]. However, these potential mechanisms have not been well examined in the context of cancer. Thus, future cancer studies need to investigate mechanistic pathways underlying the association of pain and cognition. Gaining understanding will facilitate prevention and treatments for cognitive problems among cancer survivors in pain and may also extend to non‐cancer populations.

We further found in our study that only a small percentage of the total association between bodily pain and cognitive concerns was mediated by substance use coping, while the remainder of the total association was attributed to their direct association. One possible reason for the weak mediating role of substance use coping identified in this study could be due to sample characteristics. Participants in this study reported relatively less bodily pain [40, 41] and had low variability in substance use coping scores that were screwed toward no/low substance use coping (median = 2, IQR of 2–3 for a possible raw score range of 2–8). These characteristics may explain the weak association between bodily pain and substance coping and the weak mediating effect of substance use coping in the association between bodily pain and cognitive concerns. Another possible reason could be the limitations of the measures we used for assessing pain and substance use. We used two items of pain from SF‐36, a measure designed for assessing general health condition. These two items are used and validated in cancer survivors [46, 47]; however, their use may not suffice to assess a complex pain construct. We also used two items of substance use coping as a proxy for substance use and assessed its relationship with cognitive concerns. It is possible that those who scored low on substance use coping would nevertheless consume substances to cope with pain in their lives. If that is the case, the relationship analyzed in this study could be weak, but it is possible that the nature of participants' actual relationship with substance use coping could differ if they specifically measured their use of substances rather than their substance use coping. Future studies need to include measures purported to assess the multidimensions of pain (severity and interference) and actual substance use.

Our data finds significant, yet weak relationships among bodily pain, substance use coping, and cognitive concerns, providing new insights for prevention and treatment of these conditions. This study suggests that it is crucial to develop comprehensive programs that address the multifaceted needs of cancer survivors. Rather than focusing on a single symptom and trying to treat it in isolation, it is necessary to take a broader perspective to see how the symptoms are interconnected and to base interventions on that understanding. This study also highlights the importance of pain assessment and control. If posttreatment pain is not appropriately managed, it could result in negative behavioral and cognitive outcomes. Because many cancer survivors receive inappropriate pain management [48], it is vital to examine how they cope with their pain, whether such coping is harmful, and what the consequences of that coping are. During the follow‐up cancer survivors care, it is important to screen their symptoms and provide opportunities to discuss with providers how they cope and whether there are any behavioral problems (e.g., substance use). To validate the relationship between pain and coping identified in the study, further research should be conducted among individuals experiencing moderate to severe pain and should further evaluate whether and how this relationship affects their cognitive function—not only cancer patients but also health populations. In addition, research is also needed to fully understand the motivation behind the uptake of substance use, which could help identify targets for interventions.

### 4.1 Limitations

This study has several limitations. A limitation of this study is the presence of missing data in variables such as bodily pain, substance use coping, and cognitive concerns. Approximately 6% of data were missing. However, in a large‐scale study, 6% missing data is generally considered acceptable and unlikely to meaningfully bias results [49, 50]. Future studies should aim to minimize missing data through improved data collection procedures and methods. Second, the sample included in this study had relatively good health conditions (e.g., lower bodily pain and less cognitive concerns). Further studies are needed to confirm if the associations among pain, substance use coping, and cognitive concerns exist in BCS who report higher levels of pain and cognitive concerns. Third, the measures of pain and substance use were not specific to assess the multifaceted nature of pain and current substance use. Future studies need to include measures specific to pain and substance use to understand their relationships better. Further, it would be valuable to include objective measures of cognitive function focused on specific cognitive domains (e.g., attention or memory) that may be linked to pain and substance use. Lastly, another limitation of this study is that it features a cross‐sectional design; thus, we were unable to establish temporality between pain, substance use coping, and cognitive concerns. Future studies with repeated measures will provide a more in‐depth understanding of the effect of pain on behavioral and cognitive outcomes.

## 5 Conclusion

This study highlights that higher bodily pain and higher substance use coping are associated with higher cognitive concerns among BCS, which has been an understudied area. In a clinical setting, regular assessments of pain are highly recommended, followed by appropriate treatment/intervention strategies to manage this pain (e.g., education, non‐pharmacological intervention, pain medication) [51, 52]. In particular for those reporting pain, follow‐up visits for cancer survivors would provide opportunities for clinicians to screen and/or address survivors' behavioral problems in addition to their health problems. Cancer survivors should have optimal pain symptom and behavioral control throughout their survivorship period; if inadequately treated, these can cause secondary problems. Interventions that target survivors' health needs could help reduce behavioral problems such as substance use. Further research is needed to confirm our findings through longitudinal studies that investigate motives for substance use, evaluate its relationships with pain, cognitive concerns, and related symptoms using more specific measures, and include diverse BCS with different clinical backgrounds.

## Author Contributions

**Yesol Yang:** conceptualization, investigation, writing – original draft, formal analysis, writing – review and editing, validation, visualization, methodology. **Alai Tan:** formal analysis, writing – review and editing, visualization, validation, methodology. **Leorey N. Saligan:** methodology, validation, project administration, writing – review and editing. **Diane Von Ah:** funding acquisition, writing – review and editing, data curation, supervision, methodology, project administration, validation, conceptualization.

## Conflicts of Interest

The authors declare no conflicts of interest.

## Data Availability

The data that support the findings of this study are available from the corresponding author upon reasonable request.
